# Managing the advanced cancer patient in the Australian emergency department environment: findings from a national survey of emergency department clinicians

**DOI:** 10.1186/s12245-015-0061-8

**Published:** 2015-04-29

**Authors:** Tracey J Weiland, Heather Lane, George A Jelinek, Claudia H Marck, Jennifer Weil, Mark Boughey, Jennifer Philip

**Affiliations:** Emergency Practice Innovation Centre, St Vincent’s Hospital Melbourne, Victoria Parade, Fitzroy 3065 Australia; Department of Medicine, The University of Melbourne, Parkville, 3052 Australia; Centre for Palliative Care, The University of Melbourne (St Vincent’s Hospital), Fitzroy, 3065 Australia; St Vincent’s Hospital Melbourne, Fitzroy, 3065 Australia; Erasmus Medical Centre, Rotterdam, The Netherlands

**Keywords:** Survey, Emergency medicine, Palliative care, Cancer, End-of-life care

## Abstract

**Background:**

Delivery of care to people with advanced cancer in the emergency department (ED) is complicated by competing service demands, workloads and physical design constraints. We explored emergency clinicians’ attitudes to the ED environment when caring for patients who present with advanced cancer, and how these attitudes are affected by access to palliative care services, palliative care education, staff type, ED experience and patient demographic, hospital type and region.

**Methods:**

We electronically surveyed clinicians from the College of Emergency Nursing Australasia, Australian College of Emergency Nursing and Australasian College for Emergency Medicine working in an Australian ED.

**Results:**

Respondents were 444 doctors and 237 nurses. They reported overcrowding, noise, lack of time and privacy as barriers to care. Most (93.3%) agreed/strongly agreed that the dying patient should be allocated private space in ED. 73.6% (451) felt unable to provide a desired level of care to advanced cancer patients in ED. Clinician attitudes were affected by staff type, experience, ED demographic and hospital type, but not education in palliative care.

**Conclusions:**

ED environments place pressure on clinicians delivering care to people with advanced cancer. Integrating palliative care services in ED and redesigning EDs to better match its multifaceted functions should be considered.

## Background

Emergency departments (EDs) are traditionally designed to facilitate rapid assessment, high throughput and easy physical access by clinicians to often unstable, unwell patients. They are typically comprised of open cubicle areas without solid walls, occupied by mobile trolleys rather than beds and in close proximity to one another and to staff. This design reflects the traditional prioritisation of ease of access in emergency situations, sustaining life and minimising morbidity [1] over privacy, comfort and a quiet environment. A mismatch appears to have developed between the function of EDs, with their disparate modern mandates, and their form or environment created by the traditional physical design which is geared more toward managing those with acute illness and injury than those with chronic conditions.

ED clinicians are increasingly playing a pivotal role in managing complications of chronic disease, end-of-life care (EOLC) [2,3] and the sequelae of events that precipitate death [4-6]. This is particularly apparent in the context of advanced cancer. With the increasing prevalence of cancer [7], some have suggested that there is a ‘new horizon of palliative cancer care emerging in the ED’ [8,9]. Indeed, the care of people with chronic and incurable illnesses can be expected to constitute an increasing proportion of ED work and future care planning, service development and perhaps even physical design will need to take this into account.

Patients with advanced cancer pose different management challenges to those with an isolated injury or acute medical or surgical condition. The ED and its staff are well equipped to provide lifesaving treatments but may experience obstacles to the provision of optimal palliative and EOLC [10,11]. The population of patients with advanced cancer is fragile; the burden of ill-health may be high, prognosis may be unclear, information may be lacking, and death may be imminent. People with advanced cancer may present with new symptoms, exacerbation of existing illnesses or comorbidities, complications of treatment, consequences of disease progression or difficulties with community care and support systems [12]. These factors demand an approach and an environment that balances decisive action with sensitivity, privacy and person-centredness.

Management also requires specific knowledge and skills, to allow tailoring to expected outcomes, prognosis and patient and family goals, all of which may be uncertain. The nature of the ED, however, means that such care is frequently delivered within a fast-paced, busy environment with competing service demands. Previous research in this area is limited and is typically focussed on the dying patient rather than those with advanced cancer who may have multiple ED visits prior to death.

For many patients with advanced cancer, EDs represent a portal for access to acute care, tertiary medical services and, where necessary, EOLC [13]. This is largely due to their 24-h service provision, access to analgesia and linkage to specialist and inpatient care. For some people with advanced cancer, the ED will be the location of their death [2].

Research from the US suggests that management of the advanced cancer patient in the ED is complicated by competing service demands, workloads and the physical ED design [8,11,14,15] which limits privacy [10,14]. In Australia, qualitative research revealed barriers to provision of optimal care to people with advanced cancer both within the physical environment and available resources such as inadequate time, overcrowding and access block, competition with other emergencies and lack of alternative place to provide care [8]. Limited interdisciplinary interaction and understanding was a barrier to communication and decision-making [16]. Excessive workload for nursing staff [15,17] and lack of privacy for patients and family [10,15] are reported barriers to the provision of EOLC.

A substantial body of evidence has accumulated linking factors that are increasingly endemic in EDs, such as high workloads [18], overcrowding and access block [19], to negative outcomes for all ED patients. These environmental factors, together with the unpredictable, time pressured and noisy ED environment which is busy and lacking in privacy, are likely to further complicate the provision of care to those with advanced cancer and other life-limiting illnesses. Exploring the attitudes of clinicians involved in the delivery of care to patients with advanced cancer is therefore important, since clinician perceptions may inform priorities for ED redesign.

This study aimed to add to our limited knowledge of the environmental factors that may affect Australian emergency clinicians’ attitudes to caring for patients with advanced cancer who present to an ED, in order to better prepare for the increasing numbers of these patients. Additionally, we explored how these clinician attitudes were affected by access to palliative care services, receipt of palliative care education, experience in ED, staff type, ED patient demographic, hospital type and region. Predictors of frustration in providing optimal care were also explored. ‘ED environment’ was operationalized to include factors such as physical layout, ambience, workload and system factors that affect workload.

## Methods

### Design

This was a cross-sectional survey undertaken between July and October 2012.

Inclusion criteria: We surveyed members of the College of Emergency Nursing Australasia (CENA), the Australian College of Emergency Nursing (ACEN) and the Australasian College for Emergency Medicine (ACEM) currently working in an Australian ED.

### Instruments

The survey was developed from qualitative data emerging from an associated study [8,20] by a multidisciplinary project team that included emergency physicians, palliative care (PC) physicians and researchers. Face validity of draft survey items was verified using iterative feedback between investigators. The final survey included 10 demographic items (or 9 depending on staff type) and 63 items that used Likert scales or ordinal multicategory scales to elicit a graded response. Each of these required a response for survey progression. Seven open-ended questions elicited free text responses for which completion was optional. The survey items were spread across eight domains: ED environment, clinician skills and roles, treatment, interdisciplinary collegial interaction, systems factors, views on death and dying, priorities for intervention and learning needs with further items including demographic details and access to PC services and education. The survey required approximately 15 min for completion. This paper reports results from nine Likert scale or multicategory items related to the ED environment and its relationship to provision of care for those with advanced cancer.

This study was approved by the Human Research Ethics Committee at St Vincent’s Hospital Melbourne.

### Procedure

The survey was conducted online using SurveyMonkey (SurveyMonkey.com, LLC, Palo Alto CA, USA). Eligible participants (960 College of Emergency Nursing Australasia (CENA), 256 Australian College of Emergency Nursing (ACEN) and 3,285 Australasian College for Emergency Medicine (ACEM) (1,281 emergency specialists and 2,004 trainee specialists)) were emailed an invitation, a participant information form outlining the study and participant rights, and a hyperlink to the survey.

### Data analysis

Quantitative data were exported from SurveyMonkey to SPSS 20.0 (IBM Corp. Released 2011. Armonk, NY, USA). Summary statistics (*n*, %) were calculated for each item. Percentages were adjusted to reflect varying denominators due to missed items or non-completion. Sample representativeness by region was estimated by comparing confidence intervals of sample ACEM and CENA respondents with their respective distributions for total college membership.

Comparisons were made by demographics using chi-square with adjusted residuals, linear by linear association and two-sided Fisher’s exact test for 2 × 2 contingency tables. For chi-square tests, under- or over-representativeness was assessed using adjusted standardised residuals set at ±2.0. Data for access to palliative care services were collapsed to form three independent groups for the purpose of analysis: No access or referral to offsite only, time-limited PC service or consultation and onsite access unlimited.

Binary logistic regression (enter method) was used to identify predictors of frustration at being unable to provide the care to patients with advanced cancer that they would like to provide. Based on significant univariate associations with frustration, four demographic variables and eight attitudinal variables were entered into the multivariable model. For all inferential tests, alpha was set at .05 and two-tailed tests were used.

## Results

### Demographics

The sample comprised 444 medical staff (162 trainees, 282 emergency specialists or other medical staff) and 237 nurses. The response rate amongst medical staff was 13.5% (444/3,285), while the response rate amongst nurses is unknown with uncertainty about the denominator, since survey invitations were forwarded by participants to ED nurses colleagues, and college membership is not mandatory for nursing staff. Demographic data for this sample have been reported in an earlier study from the same survey [21].

Most respondents were female (*n* = 373, 58.1%), working in an ED attended by both children and adults (*n* = 419, 65.3%) of a major referral hospital (*n* = 330, 51.4%) or major regional/rural base hospital (*n* = 166, 25.9%). Respondents were primarily from Victoria, New South Wales and Queensland (VIC: 186, 29.0%; NSW: 157, 24.5%; QLD: 142, 22.1%).

Medical respondents were highly representative of ACEM membership according to region of practice as indicated by overlapping confidence intervals for all regions except Northern Territory for which the difference was small. The regional distribution of nursing respondents is unknown for the total ACEN membership. We did not collect data on the membership status of nursing respondents; however, comparison of nursing respondents by region paralleled the regional distribution of CENA members.

### Environmental barriers to care

The majority of respondents agreed that overcrowding, noise, lack of time and privacy were barriers to care (Table 1). An overwhelming 93.3% of respondents agreed or strongly agreed that dying patients should be allocated a private space in ED (Table 1), and there were mixed attitudes regarding the perceived level of willingness of patients with advanced cancer to attend EDs. Just under two thirds felt constrained by time in caring for patients with advanced cancer; however, mixed attitudes were expressed regarding the impact of the ‘4-h rule’ [22] as part of the National Emergency Access Targets (NEAT) on assessment of patients. Only 15% agreed that the 4-h rule would make assessment of patients with advanced cancer in the ED easier, while just over half (*n* = 342, 54.3%) disagreed with this statement. Medical trainees were more likely to disagree that the new 4-h targets would make assessment of patients with advanced cancer easier (Table 2).

**Table 1:** **Summary of survey items (denominator = 629)**

	**SD**	**D**	**N**	**A**	**SA**
Overcrowding in the ED makes it an inappropriate location for patients with advanced cancer	10 (1.6)	52 (8.3)	43 (6.8)	250 (39.7)	274 (*43.6*)
The ED is too noisy to allow adequate care of patients with advanced cancer	7 (1.1)	90 (14.3)	95 (15.1)	252 (*40.1*)	185 (29.4)
The new 4-h targets will make assessment of patients with advanced cancer easier	116 (18.4)	226 (*35.9*)	187 (29.7)	85 (13.5)	15 (2.4)
There is enough time in the ED to adequately assess patients with advanced cancer	74 (11.8)	229 (*36.4*)	137 (21.8)	181 (28.8)	8 (1.3)
There is not enough time in the ED to adequately care for patients with advanced cancer	13 (2.1)	141 (22.4)	83 (13.2)	267 (*42.4*)	125 (19.9)
The ED lacks the necessary privacy to care appropriately for patients with advanced cancer	6 (1.0)	78 (12.4)	61 (9.7)	271 (*43.1*)	213 (33.9)
Access block prevents me from providing optimal care to patients with advanced cancer	5 (.8)	49 (7.8)	75 (11.9)	265 (*42.1*)	235 (37.4)
The dying patient should be allocated a space in ED that is private	17 (2.7)	7 (1.1)	18 (2.9)	153 (24.3)	434 (*69.0*)
Patients with advanced cancer are reluctant to come to the ED	20 (3.2)	127 (20.2)	267 (*42.4*)	181 (28.8)	34 (5.4)
The ED is a reasonable fall-back option for patients with advanced cancer	56 (9.1)	203 (33.1)	125 (20.4)	210 (*34.3*)	19 (3.1)
I feel frustrated that I cannot provide the care to patients with advanced cancer that I would like to provide	4 (0.7)	58 (9.5)	100 (16.3)	286 (*46.7*)	165 (26.9)

SD, strongly disagree; D, disagree; N, neutral; A, agree; SA, strongly agree. Italics represent largest response.

**Table 2:** **Attitudes of participants compared by staff type**

**Item**	**Staff type**	**Strongly disagree/neutral/disagree**	**Agree/strongly agree**	***p*** **values**
Overcrowding in the ED makes it an inappropriate location for patients with advanced cancer	Nursing	27 (11.8)^b^	202 (88.2)^a^	.002
	Medical trainees	20 (13.6)	127 (86.4)	
	Emergency specialists, other medical	58 (22.9)^a^	195 (77.1)^b^	
The ED is too noisy to allow adequate care of patients with advanced cancer	Nursing	41 (17.9)^b^	188 (82.1)^a^	<.001
	Medical trainees	52 (35.4)	95 (64.6)	
	Emergency specialists, other medical	99 (39.1)^a^	154 (60.9)^b^	
The new 4-h targets will make assessment of patients with advanced cancer easier	Nursing	181 (79.0)^b^	48 (21.0)^a^	.011
	Medical trainees	133 (90.5)^a^	14 (9.5)^b^	
	Emergency specialists, other medical	215 (85.0)	38 (15.0)	
There is no enough time in the ED to adequately care for patients with advanced cancer	Nursing	58 (23.3)^b^	171 (74.7)^a^	<.001
	Medical trainees	61 (41.5)	86 (58.5)	
	Emergency specialists, other medical	118 (46.6)^a^	135 (53.4)^b^	
The ED lacks the necessary privacy to care appropriately for patients with advanced cancer	Nursing	37 (16.2)^b^	192 (83.8)^a^	.003
	Medical trainees	34 (23.1)	113 (76.9)	
	Emergency specialists, other medical	74 (29.2)^a^	179 (70.8)^b^	
The ED is a reasonable fall-back option for patients with advanced cancer	Nursing	150 (67.0)	74 (33.0)	.028
	Medical trainees	95 (66.9)	47 (33.1)	
	Emergency specialists, other medical	139 (56.3)^b^	108 (43.7)^a^	
I feel frustrated that I cannot provide the care to patients with advanced cancer that I would like to provide	Nursing	35 (15.6)^b^	189 (84.4)^a^	<.001
	Medical trainees	44 (31.0)	98 (69.0)	
	Emergency specialists, other medical	83 (33.6)^a^	164 (66.4)^b^	

Data are number (%). ^a^Significantly over-represented according to standardised adjusted residuals; ^b^significantly under-represented according to standardised adjusted residuals.

### Access to palliative care services

Almost 80% of the participants had some onsite access to specialist PC services (*n* = 513), and a similar proportion had received education in palliative care (*n* = 78.3%) [21]. While the vast majority had some access, half of the respondents (249/496, 50.2%) reported that this was limited to standard business working hours. Just over one third (174, 35.1%) reported having an inpatient PC unit with beds onsite. Seventy-three (14.7%) reported having no access or referral to external PC services. Five respondents reported needing to access PC beds via another hospital inpatient unit rather than directly from ED in free text comments.

EDs that service the adult population only were more likely, than paediatric and mixed hospitals, to have designated PC beds onsite (adult 47.8%, paediatric 44.4%, mixed 28.8%, *p* < .001), while mixed EDs were more likely than others to have no beds onsite or have referral to offsite PC services (adult 5.1%, paediatric 0%, mixed 19.7%, *p* < .001).

### Factors affecting clinician attitudes

For all items related to environment, attitudes of clinicians did not differ significantly according to region of practice or receipt of education in PC. Attitudes were, however, affected by staff type (Table 2), years of experience working in ED, ED patient demographic and hospital type (Table 3). Nurses in general tended to have significantly greater levels of agreement, and emergency specialists greater levels of disagreement that overcrowding, ED noise, lack of privacy and time pressure are barriers to caring for patients with advanced cancer.

**Table 3:** **Attitudes of participants compared by years in ED, ED patient demographic and hospital type**

**Item**	**Years in ED**	**Strongly disagree/disagree/neutral**	**Agree/strongly agree**	***p*** **values**
Overcrowding in the ED makes it an inappropriate location for patients with advanced cancer	0 to <5	17 (13.4)	110 (86.6)	.021*
	5 to <10	18 (11.9)	133 (88.1)	
	10 to <15	23 (18.4)	102 (81.6)	
	15 to <20	20 (20.2)	79 (79.8)	
	20+	27 (21.3)	100 (78.7)	
The ED is too noisy to allow adequate care of patients with advanced cancer	0 to <5	33 (26.0)	94 (74.0)	.009*
	5 to <10	38 (25.2)	113 (74.8)	
	10 to <15	39 (31.2)	86 (68.8)	
	15 to <20	33 (33.3)	66 (67.7)	
	20+	49 (38.6)	78 (61.4)	
The new 4-h targets will make assessment of patients with advanced cancer easier	0 to <5	113 (89.0)	14 (11.0)	.036*
	5 to <10	131 (86.8)	20 (13.2)	
	10 to <15	103 (82.4)	22 (17.6)	
	15 to <20	78 (78.8)	21 (21.2)	
	20+	104 (81.9)	23 (18.1)	
There is not enough time in the ED to adequately care for patients with advanced cancer	0 to <5	49 (38.6)	78 (61.4)	.008
	5 to <10	40 (26.5)^b^	111 (73.5)^a^	
	10 to <15	52 (41.6)	73 (58.4)	
	15 to <20	36 (36.4)	63 (63.6)	
	20+	60 (47.2)^a^	67 (52.8)^b^	
The ED is a reasonable fall-back option for patients with advanced cancer	0 to <5	83 (68.0)	39 (32.0)	.002*
	5 to <10	103 (70.5)	43 (29.5)	
	10 to <15	77 (62.1)	47 (37.9)	
	15 to <20	53 (54.6)	44 (45.4)	
	20+	68 (54.8)	56 (45.2)	
I feel frustrated that I cannot provide the care to patients with advanced cancer that I would like to provide	0 to <5	32 (26.2)	90 (73.8)	.012*
	5 to <10	30 (20.5)	116 (79.5)	
	10 to <15	29 (23.4)	95 (76.6)	
	15 to <20	21 (21.6)	76 (78.4)	
	20+	50 (40.3)	74 (59.7)	
	**ED patient demographic**			
Access block prevents me from providing optimal care to patients with advanced cancer	Adult ED	55 (27.0)^a^	149 (73.0)^b^	.017
	Paediatric ED	3 (25.0)	9 (75.0)	
	Mixed ED	71 (17.2)^b^	342 (82.8)^a^	
I feel frustrated that I cannot provide the care to patients with advanced cancer that I would like to provide	Adult ED	65 (32.7)	134 (67.3)	.038
	Paediatric ED	4 (33.3)	8 (66.7)	
	Mixed ED	93 (23.1)	309 (76.9)	
	**Hospital type**			
Access block prevents me from providing optimal care to patients with advanced cancer	Major referral	81 (25.2)^a^	241 (74.8)^b^	.014
	Major regional/rural base	24 (14.9)^b^	137 (85.1)^a^	
	Urban district	20 (15.0)	113 (85.0)	
	Private hospital	4 (30.8)	9 (69.2)	
Patients with advanced cancer are reluctant to come to the ED	Major referral	220 (68.3)	102 (31.7)	.016
	Major regional/rural base	110 (68.3)	51 (31.7)	
	Urban district	80 (60.2)	53 (39.8)	
	Private hospital	4 (30.8)^b^	9 (69.2)^a^	

Data are number (%). *Linear relationship. ^a^Significantly over-represented according to standardised adjusted residuals; ^b^significantly under-represented according to standardised adjusted residuals.

Nurses and those with fewer years of experience were more likely to report that overcrowding and noise were a barrier to optimal care. Those with fewer years of ED experience were less likely to agree that the 4-h rule would make assessment of patients with advanced cancer easier (Table 3). Access block, that is, the excessive delay in access to appropriate inpatient beds due to limited health service capacity, was more likely to be viewed as an impediment to optimal care for patients with cancer by those working in a mixed ED and those who worked in a regional or rural hospital (Table 3).

Few significant attitudinal differences were present as a result of PC access; those with ‘time-limited’ access to PC services (either inpatient beds or consultative services) were far more likely to agree that access block prevents them from providing optimal care to patients with advanced cancer when compared to those with beds onsite, no access/external services only (time-limited access: 8,214/246, 87.0%; beds onsite (24-h access) 127/169, 75.1%; no access or referral to external only 56/72, 77.8, *p* = .006).

Frustration with the ability to provide optimal care was associated with attitudes to overcrowding, noise, privacy, time pressures and the belief that the dying patient should be allocated a private space in ED in the univariate analyses (Table 4). Those with time-limited access to PC services or consultation were more likely to feel frustrated with being unable to provide the level of care that they would like to compared with those that had beds onsite, but not with those that had no access or external referral only (time limited: 187/243 (77.0%), beds onsite: 107/163 (65.6%), *p* = .037).

**Table 4:** **Relationship between attitudes of participants and frustration**

		**“I feel frustrated that I cannot provide the care to patients with advanced cancer that I would like to provide”**		
**Item**	***n*** **(%)**	**Strongly disagree/neutral/disagree**	**Agree/strongly agree**	***p*** **values**
Overcrowding in the ED makes it an inappropriate location for patients with advanced cancer	Agree/strongly agree	107 (66.0)	405 (89.8)	<.001
	Strongly disagree/disagree/neutral	55 (34.0)	46 (10.2)	
The ED is too noisy to allow adequate care of patients with advanced cancer	Agree/strongly agree	76 (46.9)	348 (77.2)	<.001
	Strongly disagree/disagree/neutral	86 (53.1)	103 (22.8)	
There is enough time in the ED to adequately assess patients with advanced cancer	Agree/strongly agree	66 (40.7)	120 (26.6)	.001
	Strongly disagree/disagree/neutral	96 (59.3)	331 (73.4)	
There is not enough time in the ED to adequately care for patients with advanced cancer	Agree/strongly agree	67 (41.4)	315 (69.8)	<.001
	Strongly disagree/disagree/neutral	95 (58.6)	136 (30.2)	
The ED lacks the necessary privacy to care appropriately for patients with advanced cancer	Agree/strongly agree	92 (56.8)	383 (84.9)	<.001
	Strongly disagree/disagree/neutral	70 (43.2)	68 (15.1)	
Access block prevents me from providing optimal care to patients with advanced cancer	Agree/strongly agree	90 (55.6)	400 (88.7)	<.001
	Strongly disagree/disagree/neutral	72 (44.4)	51 (11.3)	
The dying patient should be allocated a space in ED that is private	Agree/strongly agree	143 (88.3)	429 (95.1)	.005
	Strongly disagree/disagree/neutral	19 (11.7)	22 (4.9)	
The ED is a reasonable fall-back option for patients with advanced cancer	Agree/strongly agree	81 (50.0)	148 (32.8)	<.001
	Strongly disagree/disagree/neutral	81 (50.0)	303 (67.2)	

The multivariate model, which had adequate goodness of fit (Hosmer and Lemeshow *χ*^2^ = 10.577, *p* = 2.27) and accounted for up to 34% of the variance in the model (Nagelkerke’s *R*-squared), predicted frustration with an accuracy of 79.4%. Significant predictors of frustration included having the attitude that access block prevents the delivery of optimal care, having the attitude that the dying patient should be allocated a private space in ED, and being a nurse (Table 5).

**Table 5:** **Predictors of frustration at being unable to provide optimal care to patients with advanced cancer**

	**Sig.**	**OR**	**95% CI for OR**	
			**Lower**	**Upper**
PC access, no access or referral to external PC service (ref)	.417			
PC access: time-limited service or consultation	.578	1.234	.589	2.586
PC access: PC unit with beds onsite	.709	.864	.402	1.858
Staff type, nurse	*.006*	2.275	1.271	4.073
Years experience	.092			
Years experience, 5 to < 10 years	.660	1.187	.554	2.543
Years experience, 10 to < 15 years	.773	1.121	.517	2.431
Years experience, 15 to < 20 years	.190	1.775	.753	4.183
Years experience, 20 years +	.171	.607	.297	1.240
ED patient demographic, adult (ref)	.091			
ED patient demographic, paediatric	.412	.502	.097	2.605
ED patient demographic, mixed	.064	1.648	.971	2.797
Overcrowding in the ED makes it an inappropriate location for patients with advanced cancer	.262	1.505	.737	3.073
The ED is too noisy to allow adequate care of patients with advanced cancer	.115	1.618	.890	2.941
There is enough time in the ED to adequately assess patients with advanced cancer	.320	1.333	.756	2.352
There is no enough time in the ED to adequately care for patients with advanced cancer	.118	1.549	.896	2.678
The dying patient should be allocated a space in ED that is private	*.009*	3.165	1.336	7.500
There is not enough time in the ED to adequately care for patients with advanced cancer	.174	1.527	.829	2.811
Access block prevents me from providing optimal care to patients with advanced cancer	*.000*	4.963	2.746	8.971
The ED is a reasonable fall-back option for patients with advanced cancer	.087	.648	.394	1.064
Constant	.004	.111		

Italics represent statistically significant.

## Discussion

The main purview of emergency medicine is to provide initial or definitive treatment to undifferentiated patients of various ages, diseases and injuries, to provide rapid assessment and to stabilise or resuscitate patients prior to discharge or transfer to facilities suited to their needs [23-26]. In the case of advanced cancer patients presenting to ED, these principles cannot always be adhered to; the complexity of their condition or need for sensitive communication or negotiation of treatment plans may demand more time for interaction, assessment and treatment than can reasonably be spared from competing priorities [27], resuscitative actions may be undesirable for many, in contrast to the way many other patients are treated, and finally, patients with advanced cancer cannot always be discharged from the ED [28]. Some people with advanced cancer may present at the end of their illness trajectory requiring ED clinicians to deliver EOLC [2], raising the importance of the environment in which they deliver such care.

This nationwide survey of ED clinicians reveals that significant environmental pressures within the ED, including overcrowding, noise, lack of time and limited privacy, while potentially of concern for all ED patients, are particular barriers identified by ED clinicians to the provision of quality care to people with advanced cancer. Interestingly, more experienced ED clinicians appeared less affected by the limitations of the environment in terms of noise, lack of time and overcrowding in their delivery of care to these people with advanced cancer. Similarly, they appeared to better grasp the potential benefits of improved hospital throughput and bed access potentially offered by recently introduced emergency access targets in Australia. While emergency clinicians in general have clearly articulated their concerns about these features of the ED environment [29], experience in the provision of care would appear to play an important role in helping these clinicians to find ways of circumventing these inherent environmental obstacles.

Concern about lack of privacy was the most strongly identified environmental barrier amongst surveyed clinicians. In contrast to the need for ease of access for most emergency patients, an overwhelming 93% of respondents in our survey agreed that dying patients should be managed in private rooms. A survey of patients attending an Australian ED suggests that nearly 50% of patients perceive a violation to privacy in some form and that the likelihood of experiencing a privacy violation increased with time spent in ED [30]. In a US study, the rate of overhearing conversations was similar for patients located in curtained and walled rooms. Those in curtained rooms were more likely to overhear conversations from adjacent cubicles whereas those in walled rooms were more likely to overhear conversations between staff in the hallway [31]. Data for the number of Australian EDs with a room that provides greater privacy are lacking; however, our qualitative data suggest that clinicians perceive a need in this area that is frequently unmet [8]. In overcoming the problems of limited privacy in the ED, others have addressed ED process and design to good effect [32]. In the case of patients with advanced cancer, our study has identified the pressing need for solutions to this lack of privacy.

While Australian clinicians have previously reported that their patients in the process of dying with advanced cancer should only be required to access ED and its attendant negative environmental aspects when absolutely necessary [21], results described here reveal that healthcare professionals were more ambivalent about the appropriateness of attending the ED for care for most patients with cancer, largely driven by frustration with environmental barriers to optimal care. The lack of alternative care pathways means that the ED must remain the access portal to inpatient care. Of note, a prospective study of patients with cancer with an emergency admission to a UK hospital indicated that patients preferred to be directly admitted to an oncology unit [33], whereas, in most Australian hospitals, a direct admit pathway is not readily available.

Effective emergency care for any patient necessitates the marriage of ED design to ED purpose, processes and operation [34]. Before issues of design can be addressed, however, we must first address issues of function. There is considerable heterogeneity in the functions and forms of EDs worldwide. This is apparent in terms of the patient characteristics (children, adults, mixed), the presence of streaming for certain conditions, private versus public, contiguous versus non-contiguous layout, functional units for disease-related groups, streaming of patients depending on acuity and anticipated disposition and clinic areas for minor complaints. When functional requirements are in direct competition with one another, it is reasonable to assume that provision of optimal care may be strained or compromised.

The increasing number of older, sicker patients, including those with advanced cancer, points to the urgent need for EDs to use data-driven processes to expedite care. To counter problems of access block and ED overcrowding, ‘Lean Thinking’ [35] methodologies have been applied in the ED context both in Australia [36] and internationally [35]. Lean principles focus on improving efficiency by minimising the time and resources required to provide services, eliminating practices that do not value-add and increasing those that do. This approach has produced a range of positive outcomes in terms of waiting times, ED length of stay and the proportion of patients that do not wait [29,36]. It remains to be seen, however, whether the burgeoning number of older, sicker patients with chronic illness including cancer will be adequately managed by such a system, and whether such system improvements can withstand being overwhelmed by the growing caseload. It is clear that lean principles must take into account the need for environmental redesign that matches the changing demographic. Recent efforts at redesign have focussed largely on processes within the ED. Such efforts, however, may be futile in facilitating throughput if congestion amongst inpatient units remains, with hospitals continuing to operate at 100% or higher bed capacity. To prevent any benefit of ED redesign being negated by shortfalls in hospital beds, a ‘whole of hospital’ approach to redesign is necessary. Patients that are most affected by access block and ED overcrowding are those for whom a hospital admission is unplanned due to their medical conditions [19,29,37].

Effective redesign must extend beyond the hospital too. There is increasing evidence of the benefits of earlier referral to palliative care services, including fewer ED presentations. For example, earlier referral to PC services has been shown to reduce ED presentations amongst both adult and paediatric patients when compared to usual care [20,38]. In advanced lung cancer, early PC consultation not only results in fewer ED presentations and hospitalisations but also improved survival when compared to their more aggressively treated counterparts [39]. Increased PC resources for people with advanced cancer and earlier referral, with integration of PC alongside anticancer treatments, may well also reduce ED presentations in other cancer types. Various models have emerged including cancer-specific EDs [33], and models that integrate PC services within general EDs or that provide a PC service in parallel to the ED [34]. Certainly improved communication and collaboration between oncology, PC and ED staff when patients with advanced cancer attend the ED can only improve their care. Given that 60% of the participants surveyed felt that they did not have sufficient time to care for an advanced cancer patient, the use of dedicated PC staff integrated into the ED may, in some instances, be an important way of achieving optimal quality of care.

The vast majority of respondents agreed to feeling frustrated in being unable to provide the desired level of care to patients with advanced cancer. Several attitudinal factors were related to feeling frustrated including that access block prevented the delivery of optimal care, having the belief that the dying patient should be allocated a private space in ED, and being a nurse. It is unsurprising that nurses may experience more frustration given that they may be likely to spend more time providing direct care to patients.

Views were mixed about the ED being an ‘appropriate fall back option’ for people in advanced cancer. Those more likely to agree with this sentiment of the ED being appropriate were emergency specialists and other medical staff and those with more experience. Respondents who felt frustrated in care provision were more likely to disagree with the ED being a suitable option for patients with advanced cancer.

### Limitations

This study is limited by the modest response rate. For nurses, the response rate was unknown, but for doctors, a response rate of 13.5% was obtained. This is comparable to other studies surveying this population using similar methods [40,41]. This may represent ‘survey fatigue’ in the target group. While low response rate does not necessarily reduce representativeness [42], caution should be taken when generalising beyond the sample of this study. The fact that no denominator could be calculated for the nursing proportion of the sample makes assessing external validity difficult. Further, the low response rate for the medical proportion of the sample that was obtainable raises the issue of responder bias, that is, respondents may have been more likely to participate if they had a particular interest or experience in treating patients with advanced cancer. There is, however, some empirical evidence against major response bias, as our sample of doctors and nurses was similar in terms of regional distribution of doctors and nurses that are members of ACEM and CENA, respectively. Our use of web-based technology to survey clinicians may have resulted in selection bias against those with limited internet access or for whom the use of the internet is undesired. We attempted to minimise measurement bias by establishing face validity and content validity of the tool, given the absence of a previously validated survey on managing patients with advanced cancer in ED. Finally, there is a possibility that this study was subject to type 1 error due to the number of comparisons made. The large effect sizes found across much of our data, however, suggest that we can confidently conclude that true differences exist between groups compared.

## Conclusions

Care of patients with advanced cancer in the ED environment is clearly challenging. Clinicians perceive that environmental barriers negatively affect the care they can provide, specifically overcrowding, noise, lack of time and privacy. A one-size-fits-all approach to care in the ED is not appropriate for the advanced cancer patient and indeed unlikely to suffice for the predictions of increasing elderly and multicomorbid patients attending ED in the future. The delivery of sensitive, person-centred care is critical for these vulnerable patients, and our learnings elsewhere suggest that such work is perceived as both desirable and satisfying by clinicians [21]. Yet, this care does require flexible models of care with capacity for greater time to be spent with patients and families to avoid frustration and dissatisfaction. Consideration should be given to the better integration of PC services in the ED and to better matching ED design to its various functions.
